# Locating the Association

**Published:** 1885-09

**Authors:** R. J. Nunn

**Affiliations:** Savannah, Ga.


### LOCATING THE ASSOCIATION.

*Editors Atlanta Medical and Surgical Journal:*

The arguments which have been or may be urged in favor of locating the Association are among others, perhaps,

1. "Better preservation of its archives."

2. The more prompt and better editing and publication of its papers.

3. The more regular conduct and dignity of its proceedings.

4. Having a hall in which to meet at any time in emergency and in which committees could meet during recess.

5. Having a library.

6. Having a museum.

*1st.—"Better Preservation of its Archives."*

The archives of the Association are or should be its printed minutes, etc. The manuscripts from which these are printed can be of no earthly value after the printer is through with them, unless, indeed, they might become valuable as curiosities if preserved for a century or two. It is not, however, very clear how this better preservation of what we may for the moment dignify by the name of archives is to be effected. They would still be confided to the keeping of some one of the officials of the Association to be kept at his private residence and handed over to his successor in office, so that, unless the Association can provide a suitable place in which to preserve its archives, and also elect an official to reside there and take charge of them, the preservation of these historical curiosities would not be one whit better than at present.

It must be observed that a scientific body like the Medical Association of Georgia, with its printed proceedings, which are its record, stands to its manuscript archives in a very different relation to that occupied to them by bodies whose only records are their written minutes.

*2d.—The More Prompt and Better Editing and Publication of Its Papers.*

It is difficult to see in what way the location of the place of meeting of the Association can effect the "editing and publication of its papers." The facilities of publication depend rather upon the location of the printing office doing the work and the residence of the secretary of the Association, who is ex-officio chairman of the Committee on Publication, or rather it may be said, to narrow itself down to a question of the cost of express charges or postage incurred in the transmission of "copy and proof," the time required to accomplish their interchange is so trifling that it may well be ignored as having little or no effect upon the date of publication of the Transactions.

*3.—The More Regular Conduct and Dignity of its Proceedings.*

It is to be presumed, of course, that the officers of the Association would be selected in the same manner as at present and would bring to the office each his individuality, his fitness or unfitness for the proper and dignified discharge of the duties of the position he would hold, and this, the personality of the officer, could, in no way, be affected by the location of the city in which the Association may chance to meet.

*4th.—Having a Hall in which to Meet at any Time in Case of Emergency, and in which Committees could meet During Recess.*

It is hardly necessary to point out that this is impracticable and unnecessary. Impracticable because the Association has no funds to invest, and unnecessary because as a scientific body there can be no such thing as meetings of emergency. Finally, committees could most likely meet at whatever point would be most convenient to the majority of their members should they by any chance hold meetings during recess.

*5.—Having a Library.*

Such a library would be of value to the local profession only. It certainly could not be of any use to the general membership of the Association, who would have but little time or inclination for medical reading during three days of the session of the Association.

It is quite true that the library might be a circulating one, but in this day of cheap medical literature every practitioner will prefer to have his books at hand in his own library for speedy reference in case of necessity.

Another and serious objection to this phase of the scheme is that the Association has no money to make such an investment as would be worthy of the name of library.

Finally, the establishment of a library carries with it the creation of the official having charge thereof, who would require to be paid for his services and should also be permanently located.

Altogether, this portion of the scheme appears to be at present impracticable.

*6.—Having a Museum.*

The acquisition and maintenance of a museum by the Association as at present organized is impracticable for the self-same reasons that apply to the library; moreover, it would, if possible, be even less useful. Specimens could not be circulated. Pathological preparations cannot be bought, even if the Association had money to invest in them. They can only be acquired by donation and gradual accumulation, and their preservation demands constant and skilled attention, again implying the necessity for a resident salaried official.

*Let us now glance at the inconveniences and disadvantages which would result from a permanent location of the Association:*

1. As of course the secretary would have charge of the archives, it would be a matter of necessity that he should be permanently located at the place of meeting.

2. The secretary being ex-officio chairman of the "Committee on Publication," it would soon be found imperatively necessary that at least two members thereof should likewise reside at the place of meeting, to the end that the committee would be always ready for work. This would be but the point of the wedge, and soon all the officers would be selected from the central point.

3. The members living at a distance would find it inconvenient to spend a long time and much money in traveling to and frcm the place of meeting every year. The burden would fall heavily upon the many members living throughout the State, while the few residing at the place of meeting would be correspondingly convenient. As a consequence, the attendance of members from a distance would gradually diminish. This would be followed by a loss of interest in the doings of the Association and a withdrawal from membership, and soon the Association would be composed only of those physicians who clustered about the central point. It would become, in fact, merely a local society.

The fate of the Georgia Medical Society is an example of this. Chartered in 1804, its name would indicate that it was intended by its founders to be a State institution, but the mistake was made of locating it in Savannah, and, as a natural consequence, it has become a local society.

4. Located in one place, the votes of the local profession would soon outweigh that of the rest of the State, and in matters of legislation, or recommendations for the direction of legislation, this might become a serious matter in cases where local interests might clash with those of the profession at large. For this reason it would be unwise to meet regularly at any one place, and would be much more prudent to hold the meetings of the Association at the same place only at irregularly recurring periods, and at distant intervals of time.

### *Peripatetic Medical Societies.*

It is unnecessary to multiply arguments to demonstrate the superiority of the migratory system of holding meetings; the crucial test of actual experience witnesses strongly in its favor.

The British Medical Association, the largest and most influential medical society in the world, is a peripatetic body.

The American Medical Association is also a migratory body, and if we would draw examples outside the medical profession, we would find that many of the most powerful scientific and other bodies are peripatetic as to their meetings.

The International Medical Congress is a similar body, covering greater areas, and calculated to embrace the world.

These great bodies evidence the value of the system, when worked upon the grandest scale, but I have before me the results of the same principle applied to a local medical society.

Nearly a score of years ago the Georgia Medical Society (the local society of Savannah) adopted the plan of meeting weekly at the houses of the members, and has continued the custom to the present time. Every regular practitioner in the city appears upon its roll of membership, and I challenge the State or the United States to produce a more loyal, ethical, united or brotherly local profession than the one of which we in this city are so proud, and this result is, without dissent, attributed to our weekly peripatetic meetings, because previous to the adoption of that plan it was almost a matter of impossibility to get a meeting of the Society.

These are the extremes, the army corps and the company. It cannot be possible that the principle of conduct, which has proved successful in these two, should be applicable to the intermediate bodies, the battalion or the brigade.

There is no place where men can be known as well as in their own homes, and so let the seaboard go to the mountains, and let Cherokee visit the wire-grass, and let us see one another among our household gods. We will be much better friends for this social intercourse. Surely we feel much more friendly to a man when we can walk up and take him by the hand, and say I met you at Macon, or I owe you much for a suggestion I heard from you at Augusta, and I remember, with pleasure, your humorous remarks at Atlanta. Let us not be deprived of this pleasure.

If I plead in favor of the peripatetic meetings, I do so in the interest of the poorer and the more hard-worked members of the profession, many of whom cannot afford the time or money to attend the meetings of the Association, but who would be glad to see us at their homes, if it could be accomplished without too great a tax upon their limited resources. If these men cannot come to us, we can and ought to go to them.

*Journalizing the Proceedings.*

Beyond a doubt the phenomenal success of the British Medical Association has been due in a great measure to the establishment of the *British Medical Journal*, the weekly organ of the Association, and it is almost certain that the result will be similar in the case of the American Medical Association and its Journal.

The march of science is much too rapid to await the slow, cumbrous method ot getting out a volume of "Proceedings" once a year. The novelty and snap have worn off the papers by the time they are printed, and their value becomes more historical than suggestive.

With such views it will easily be understood that I, for one, hailed with pleasure the idea of printing the proceedings of the Medical Association of Georgia in the form of a monthly journal. I certainly thought it would give a renewed impetus to the Association, which would result in an increase of membership and a more widespread influence, and I had no idea that there would be any conflict of interests in such a course as there now seems to be.

This matter is mentioned here simply to show that it has not escaped my attention, but I will reserve the discussion of this subject to a more appropriate occasion.

*Proportion of the membership of the Association to the profession of the State, compared with the roll of the American Medical Association in comparison with the number of practitioners in the United States:*

A Northern medical journal expressed its astonishment some weeks ago at the small number of members in the Medical Association of Georgia, when compared with that of the profession throughout the State. In 1884 the membership of the Association was 272 (see Proceedings 1884), the number of physicians in the State the same year was 1,725, (see *Georgia State Gazettteer and Directory 1883-84*) showing that a little over 15 per cent. of all who claimed the name of doctor were then members of the regular State organization. What deduction should be made from the State figures for irregulars of various schools, I am unable to say.

According to the United States Census 1880, the number of physicians in the United States was 85,671, and the membership of the American Medical Association, as given in its Transactions for 1881, was 2,374, being about 2.7 per cent.

It certainly seems that the Medical Association of Georgia makes a very good showing in this comparison, and that its hold upon the profession of the State is not quite so bad as some of our Northern friends would have us believe.

*A Weak Point in the Present Mode of selecting a Place of Meeting.*

As will be seen farther on, there are a number of towns quite large enough to give the needed hotel accommodations for the meetings of the Association and having a profession sufficiently numerous to make it to the interest of the Association to meet in these places, but from many of these it is practically excluded because the Association has little, or perhaps no membership in these places, and hence there is no one to tender to the Association an invitation to hold its meeting there, much less to bear the expense of the meeting and of an entertainment, which, by custom, has come to be expected.

Respectfully,

R. J. NUNN, M. D.

SAVANNAH. August, 1885.

